# Electrochemical Behavior and Cytocompatibility of Titanium Dental Implants Under Different Chemical Treatments

**DOI:** 10.3390/biomedicines13102457

**Published:** 2025-10-09

**Authors:** Alexandra-Camelia Pogacian-Maier, Radu Septimiu Campian, Alexandru Mester, Marioara Moldovan, Ioan Petean, Emoke Pall, Simona Varvara, Andra Piciu, Dragos Ene

**Affiliations:** 1 Department of Oral Health, University of Medicine and Pharmacy “Iuliu Hatieganu” Cluj-Napoca, 400012 Cluj-Napoca, Romania; 2 Doctoral School, University of Medicine and Pharmacy “Iuliu Hatieganu” Cluj-Napoca, 400347 Cluj-Napoca, Romania; 3 Institute of Chemistry “Raluca Ripan”, University Babes-Bolyai, 400294 Cluj-Napoca, Romania; 4 Faculty of Chemistry and Chemical Engineering, Babes-Bolyai University, 400084 Cluj-Napoca, Romania; 5 Department of Infectious Diseases, Faculty of Veterinary Medicine, University of Agricultural Sciences and Veterinary Medicine Cluj-Napoca, Calea Mănăştur nr. 3-5, 400372 Cluj-Napoca, Romania; 6 Department of Cadastre, Civil and Environmental Engineering, Faculty of Economic Sciences, “1 Decembrie 1918” University of Alba Iulia, 15–17 Unirii Street, 510009 Alba Iulia, Romania; 7 Department of Medical Oncology, University of Medicine and Pharmacy “Iuliu Hatieganu” Cluj-Napoca, 400012 Cluj-Napoca, Romania; 8 Department of General Surgery, Faculty of Medicine, University of Medicine and Pharmacy Carol Davila Bucharest, 050474 Bucharest, Romania; 9 Bucharest Emergency University Hospital, 050098 Bucharest, Romania

**Keywords:** dental implant, titanium implant, peri-implantitis, osseointegration, decontamination

## Abstract

**Background:** This study aimed to evaluate the impact of different chemical treatments on titanium implant surfaces and their biological compatibility. **Methods:** Titanium dental implants were immersed in Ringer’s solution, hydrogen peroxide (3%), citric acid (40%), EDTA (40%), or a citric–phosphoric acid mixture. Electrochemical behavior was analyzed using open-circuit potential monitoring and electrochemical impedance spectroscopy over 168 h. Cytocompatibility was assessed by culturing human gingival mesenchymal stem cells (MSCs) directly on treated implants and in conditioned media, followed by viability evaluation through CCK-8 assays. **Results:** Citric acid and Ringer’s solution preserved passive film stability and supported high MSC viability (>75%) with minimal cytotoxic effects. Hydrogen peroxide and the citric–phosphoric acid mixture caused pronounced surface corrosion, decreased impedance stability, and significantly reduced cell viability (57–65%). EDTA-treated surfaces showed intermediate results, with moderate viability but impaired cell adhesion. **Conclusion:** The findings highlight the dual influence of chemical decontamination on implant stability and biological response. Citric acid and Ringer’s solution appear to be safer protocols for surface decontamination, whereas hydrogen peroxide and mixed acid treatments should be applied with caution due to their detrimental electrochemical and cytotoxic effects.

## 1. Introduction

Dental implants have become a widely accepted solution for the rehabilitation of partial and full edentulism, providing functional and esthetic improvements with long-term success rates [1,2]. However, despite these high success rates, implant failures do occur and are often associated with peri-implant diseases [3]. One of the key challenges in the management of these conditions is the effective decontamination of the implant surface to eliminate bacterial biofilms and restore a biocompatible surface that promotes re-osseointegration [4].

Implant surface decontamination is a critical step in the treatment of peri-implantitis [5,6]. Various mechanical, chemical, and laser-based techniques have been employed for this purpose, each with specific advantages and limitations [5,6]. Mechanical decontamination methods, such as air abrasion and ultrasonic scaling, are effective when removing biofilm, but may alter the implant surface topography, potentially compromising re-osseointegration [7]. Chemical agents, including chlorhexidine, hydrogen peroxide, and citric acid, have been widely studied as adjunctive therapies for their antimicrobial and detoxifying properties [8,9]. However, the effects of these chemicals on implant surfaces are not fully understood and remain a subject of ongoing research.

Surface characteristics play a pivotal role in implant success by influencing cellular adhesion and the integration process [10]. Consequently, it is crucial to evaluate how chemical decontamination protocols affect these surface properties. While chemical agents may offer effective antimicrobial action, there is a growing concern that prolonged exposure or high concentrations could alter the surface topography or chemical structure of the implant, potentially reducing its osteoconductive properties [11].

Based on the existing literature, we hypothesized that: (a) citric acid would preserve surface passivity and cytocompatibility due to citrate-mediated oxide stabilization; (b) Ringer’s solution would maintain stability as a neutral irrigant; (c) hydrogen peroxide and the citric–phosphoric mixture would compromise electrochemical stability and reduce cytocompatibility; and (d) EDTA would yield intermediate effects due to its chelating properties.

This in vitro study aims to evaluate the impact of various chemical agents on titanium implant surface decontamination, and analyze their effects on surface morphology, chemical composition, and biocompatibility. By comparing different chemical decontamination protocols, the study seeks to provide evidence-based recommendations for clinicians in managing peri-implantitis while preserving implant surface integrity and promoting re-osseointegration.

## 2. Materials and Methods

### 2.1. In Vitro Experiment Protocol

In this in vitro study, we used 20 dental implants made of Ti6Al4V alloy (B&B Dental Implant, Bologna, Italy). The implants were immersed in the following chemical agents: Ringer solution; hydrogen peroxide (3%); citric acid 40% (Cerkamed, Stalowa Wola, Poland); Ethylenediaminetetraacetic acid (EDTA) solution 40% (Cerkamed, Stalowa Wola, Poland), mixture (1:1) of citric 2% and phosphoric (1N, Merk) acids, artificial saliva (0.4 g/L NaCl, 0.4 g/L KCl, 0.795 g/L CaCl_2_·2H_2_O, 0.78 g/L NaH_2_PO_4_·2H_2_O, and 1.0 g/L urea, adjusted to pH 6.8). A 7-day immersion period was selected for electrochemical measurements, in order to capture the long-term stability and degradation kinetics of the passive oxide film under continuous exposure. For each experimental group, four implants were immersed individually in 10 mL of the respective chemical solution, maintained at room temperature (22–25 °C) in sterile glass containers. After this period of immersion, specimens were removed from the solutions, rinsed with sterile saline, and dried for further analysis.

### 2.2. Cytocompatibility Assessment of Chemically Conditioned Dental Implant Surfaces Using Human Gingival Mesenchymal Stem Cells

To evaluate the potential cytotoxic or inhibitory effects of dental implants conditioned in different chemical environments, a standard human gingiva-derived mesenchymal stem cell (MSC) line was used. These cells were functionally and immunophenotypically characterized according to established criteria. Biomaterial samples (dental implants) were conditioned by immersion in one of five test solutions: hydrogen peroxide, citric acid, citric and phosphoric acid, Ringer solution, or EDTA.

A 48 h conditioning period, at room temperature, was chosen for cytocompatibility assays, as prolonged exposure would compromise cell viability in vitro, and thus, this duration best reflects acute cellular responses to chemically treated implant surfaces. After conditioning, the implants were transferred into 24-well tissue culture plates. A suspension of 1 × 10^5^ MSCs was seeded directly onto each biomaterial. Cultures were incubated at 37 °C in a humidified 5% CO_2_ atmosphere. After 30 min, DMEM/F12 (Lonza) medium supplemented with 15% fetal calf serum (Sigma-Aldrich, Burlington, MA, USA) and 1% antibiotic–antimycotic (Sigma-Aldrich, Burlington, MA, USA) was added to each well. After 24 h of incubation, cell viability was assessed using the CCK-8 assay. To assess indirect cytotoxicity, conditioned media (the five original solutions in which the biomaterials were incubated) were also tested. MSCs were seeded into standard 24-well plates, allowed to adhere for 24 h, and then exposed to a 1:1 mix of propagation medium and conditioned medium for an additional 24 h. Following all incubations, 10 µL of CCK-8 reagent (Sigma-Aldrich) was added to each well and incubated for 4 h at 37 °C in the dark. The formazan product formed by metabolically active cells was quantified by measuring absorbance at 450 nm, using a microplate reader (Bio-Rad, Hercules, CA, USA). All conditions, including the untreated control group (cells cultured in standard medium), were tested in triplicate. Cell viability in the untreated control group was defined as 100%, and the values of all experimental groups were expressed as percentages relative to this baseline.

### 2.3. Open-Circuit Potential and Impedance Spectroscopy Protocols

Electrochemical experiments were conducted in a conventional three-electrode glass cell (100 cm^3^ capacity) using a PARSTAT 2273 potentiostat/galvanostat (Princeton Applied Research, Ametek, Berwyn, PA, USA). A platinum foil and an Ag/AgCl/KCl_sat_ electrode served as the counter and reference electrodes, respectively. Prior to testing, the implants were cleaned with ultrapure water, dried at room temperature, and insulated with Parafilm, leaving a constant 7 mm length exposed to the electrolytes. This approach ensured a consistent exposed surface area throughout all experiments.

The implants were immersed in the test solutions for about 1.5 h to attain a steady-state condition, and the open-circuit potential (OCP) values were recorded every second during this period. Additionally, OCP values were measured prior to each electrochemical impedance spectroscopy test conducted at various immersion times. All open-circuit potential values are referenced as Ag/AgCl/KCl_sat_.

The EIS measurements were performed over a period of 168 h of continuous immersion. EIS spectra were recorded every 15 min during the initial 8 h of immersion in the solutions and subsequently every 24 h. EIS measurements were obtained in the frequency range of 10 kHz–10 mHz with five points per Hz decade, using a 10 mV sinusoidal perturbation around the OCP.

### 2.4. Scanning Electron Microscopy (SEM)

The microstructure of Ti6Al4V alloy dental implants was investigated with a Hitachi SU8230 (Hitachi Company, Tokyo, Japan), operated at 15 kV in high vacuum mode. The backscattered electron images (BSE) were taken, and the EDS (Energy Dispersive Spectroscopy) elemental spectra were taken for the relevant grains. The elemental investigation was carried out with a X-Max 1160 EDX elemental probe cooled with liquid nitrogen (Oxford Instruments, Oxford, UK).

### 2.5. Statistical Analysis

All experimental conditions were tested in triplicate, and quantitative results were expressed as mean ± standard deviation (SD). Statistical analysis was performed using one-way analysis of variance (ANOVA) to detect significant differences in cell viability across the different treatment groups. The coefficient of determination (R^2^) was calculated to assess the proportion of variability explained by the treatment conditions. To evaluate the assumption of homogeneity of variances, the Brown–Forsythe test was applied. Post hoc pairwise comparisons were performed using Tukey’s test to adjust for multiple comparisons. A *p* value under 0.05 was used for all statistical tests.

## 3. Results

### 3.1. Proliferation and Morphology of Mesenchymal Stem Cells

**Figure 1:**
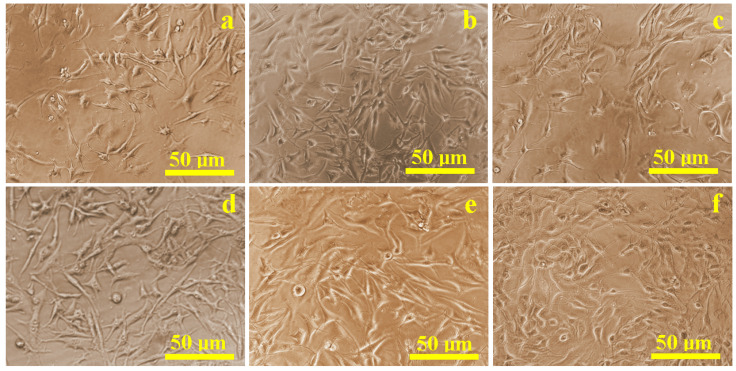
Optical microscopy images of the cell proliferation on the presence of the corrosive solution: (**a**) control sample, (**b**) Ringer’s solution, (**c**) hydrogen peroxide, (**d**) citric acid, (**e**) EDTA, and (**f**) mixture of citric and phosphoric acids.

The proliferation of mesenchymal stem cells (MSCs) cultured in flat-bottom wells with conditioned media from the different treated implants was evaluated using biological optical microscopy (Figure 1). Most samples, except those treated with hydrogen peroxide, exhibited predominantly healthy cells with rounded nuclei measuring 10–15 µm, and well-developed cytoplasmic projections between 15 and 20 µm. Cell density on all surfaces was comparable to the control sample (Figure 1a), except for the hydrogen peroxide group (Figure 1c). Several cells showed compromised morphology, including disrupted membranes and displaced nuclei, consistent with cell death, in addition to reduced overall density.

### 3.2. In Situ Cellular Behavior on Titanium Surfaces

**Figure 2:**
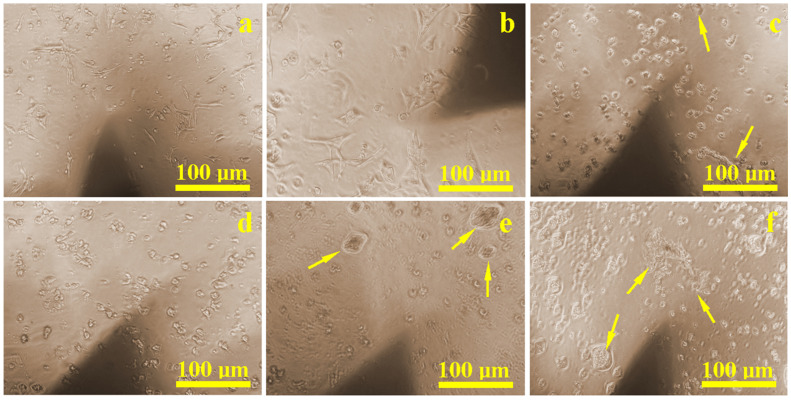
Optical microscopy images of the cell proliferation on the implant surface: (**a**) control sample, (**b**) Ringer’s solution, (**c**) hydrogen peroxide, (**d**) citric acid, (**e**) EDTA, and (**f**) mixture of citric and phosphoric acids. Yellow arrows indicate clusters of corroded material with an inhibition zone around them where the cell density is lower.

During the culturing period, in situ inspections revealed that the control sample (Figure 2a) had a dense and uniform distribution of cells that were well-attached through developed extensions. Similar results were found on surfaces treated with Ringer’s solution (Figure 2b) and citric acid (Figure 2d), where healthy cell populations were present and only slightly reduced compared to the control. Surfaces treated with hydrogen peroxide (Figure 2c) and EDTA (Figure 2e) showed localized corrosion areas, accompanied by visibly lower cell density. The weakest proliferation was observed on the sample etched with a mixture of citric and phosphoric acid (Figure 2f), where corrosion debris created visible inhibition zones. However, in areas free of corrosion debris, normal cellular development and density were still observed.

### 3.3. Cytotoxicity Assessment

**Figure 3:**
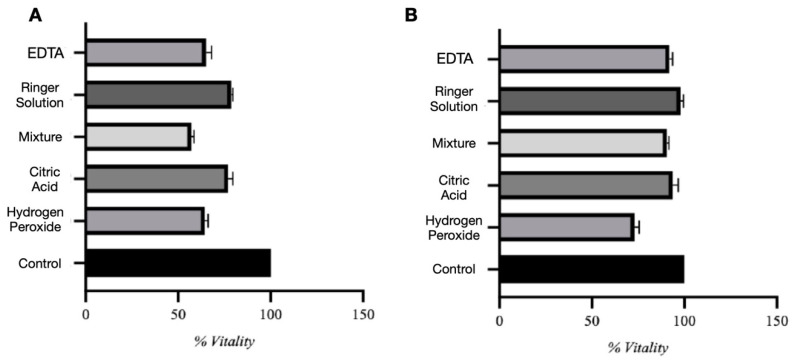
Direct (**A**) and indirect (**B**) assay evaluation.

Viability data are presented as percentages normalized to the untreated control group, which was defined as 100%. In the direct contact assay (Figure 3A), the control group consistently showed 100% cell viability. Among experimental groups, biomaterials conditioned in Ringer solution and citric acid demonstrated the highest viability, averaging 78.7% and 77.1%, respectively. Despite a moderate decrease compared to controls, these conditions showed relatively low cytotoxicity. However, many viable cells were observed to be floating in the culture medium rather than adhering, suggesting impaired cell–substrate interaction. Biomaterials conditioned in hydrogen peroxide and EDTA showed significantly lower viability, around 64–65%, while citric and phosphoric acid demonstrated the most substantial cytotoxic effect, with viability dropping to 57.1%. One-way ANOVA showed significant differences between all test groups (F = 196.0, *p* < 0.0001), with a high R^2^ of 0.9879, indicating that variability was largely explained by the treatment conditions. The Brown–Forsythe test revealed no significant variance differences between groups (*p* = 0.5116), confirming the reliability of the ANOVA. Tukey’s post hoc analysis confirmed significant differences between all groups (*p* < 0.05).

In the indirect assay (Figure 3B), using conditioned media, the control group maintained 100% viability. Conditioned media from Ringer solution, citric acid, citric and phosphoric acid, and EDTA all preserved high MSC viability (90–99%), indicating minimal release of toxic substances. In contrast, media conditioned with hydrogen peroxide reduced viability to approximately 73%**,** suggesting the release of cytotoxic compounds. Statistical analysis confirmed significant differences among groups (F = 74.92, *p* < 0.0001, R^2^ = 0.9690), and the Brown–Forsythe test again showed no significant differences in variance (*p* = 0.5754).

### 3.4. Open-Circuit Potential Measurements

**Figure 4:**
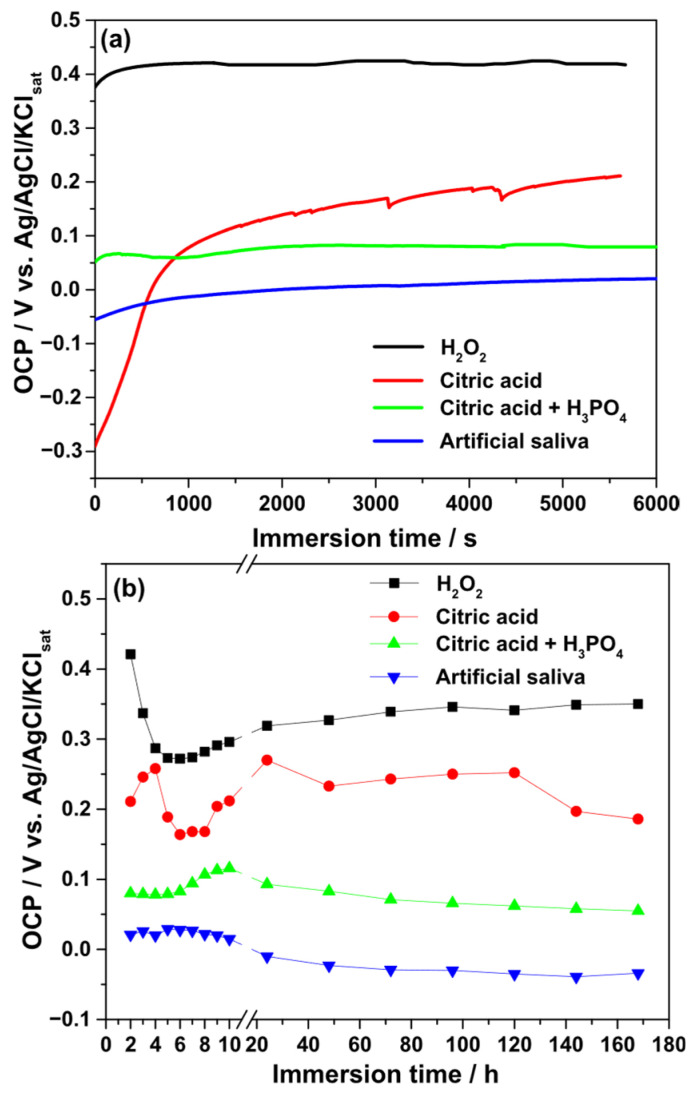
Variation in the OCP values during the continuous immersion of Ti–6Al–4V alloy samples in different electrolytes. (**a**) Short-time measurements (1.5 h); (**b**) long-term measurements (168 h).

The evolution of the OCP was monitored during both short-term (1.5 h) and long-term (168 h) immersion of Ti–6Al–4V implants in different electrolytes. Figure 4 illustrates the time-dependent variation in OCP values. For the 3% H_2_O_2_ solution, the OCP rapidly increased to a noble potential of approximately +0.42 V (vs. Ag/AgCl/KClsat), then decreased to +0.27 V during the first 5 h of immersion. Afterward, the OCP gradually increased, stabilizing around +0.346 V by 168 h.

In 40% citric acid, the OCP increased from −0.30 V to +0.20 V within the initial 1.5 h, indicating film stabilization. During prolonged exposure, transient reactivity was observed within the first 48 h, after which the OCP stabilized near +0.25 V. At the end of the test, the value decreased slightly to +0.186 V. Exposure to the citric–phosphoric acid mixture showed initial instability during the first 0.5 h, followed by stabilization at +0.08 V after 6 h and a gradual increase to +0.116 V at 10 h. Extended immersion caused a slow decrease in OCP values.

In artificial saliva, the OCP increased from −0.055 V to +0.22 V during the first 10 h, after which a gradual decrease toward −0.03 V was recorded. Overall, the relative stability of the passive film, as suggested by OCP measurements, decreased in the following order: H_2_O_2_ > citric acid > citric–phosphoric acid mixture > artificial saliva.

### 3.5. Electrochemical Impedance Spectroscopy

**Figure 5:**
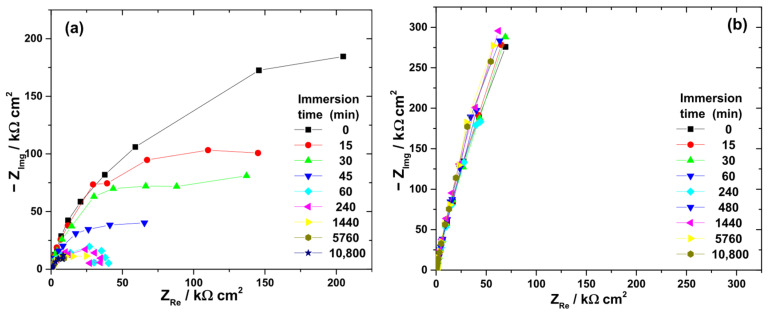

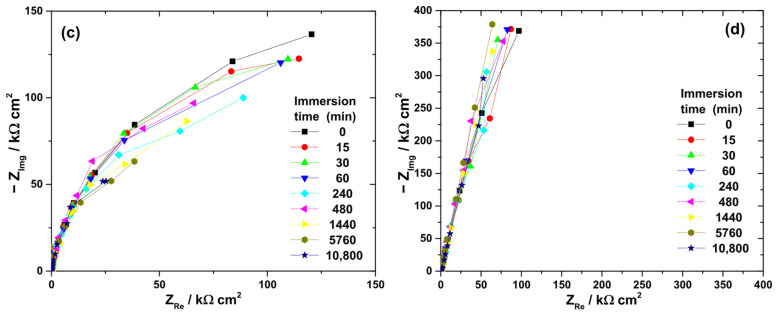
Typical Nyquist diagrams obtained at OCP during the immersion of Ti-6Al-4V implant samples for various periods of time in different electrolytes: (**a**) 3% H_2_O_2_ solution; (**b**) 40% citric acid solution; (**c**) mixture of citric and phosphoric acids; and (**d**) artificial saliva.

**Figure 6:**
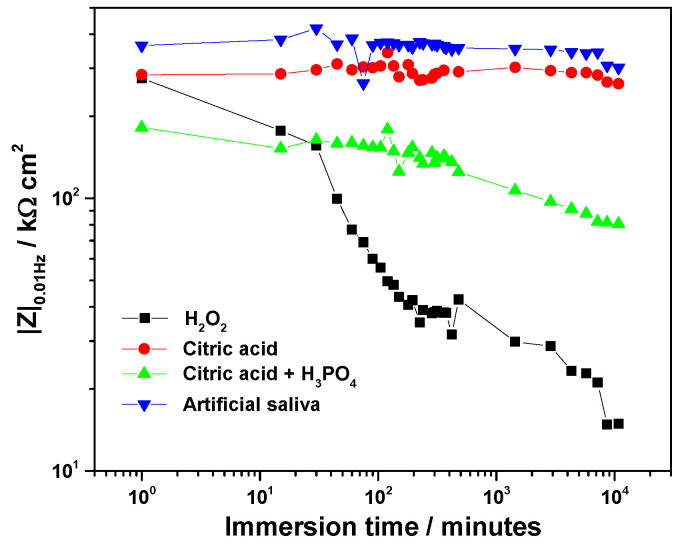
Time-evolution of the low-frequency impedance modulus, |Z|_0.01_Hz for Ti-6Al-4V implant exposed to various electrolytes.

Nyquist diagrams collected during immersion in the four electrolytes (Figure 5) displayed capacitive behavior, consistent with passivated surfaces. The low-frequency impedance modulus (|Z|_0.01_Hz) was used as an indicator of passive film resistance (Figure 6).

At the beginning of immersion, high |Z|_0.01_Hz values confirmed the effective barrier properties of the native oxide. The highest initial value was recorded in artificial saliva (363 kΩ·cm^2^), followed by citric acid (284 kΩ·cm^2^), H_2_O_2_ solution (276 kΩ·cm^2^), and the citric–phosphoric mixture (182 kΩ·cm^2^).

Significant electrolyte-dependent changes were observed during prolonged immersion. In the 3% H_2_O_2_ solution, the |Z|_0.01_Hz value decreased sharply to 99.7 kΩ·cm^2^ within the first 45 min and continued to decline, reaching 14.9 kΩ·cm^2^ after 168 h. For the citric–phosphoric acid mixture, |Z|_0.01_Hz showed a monotonic decrease throughout the exposure period, attaining 80.6 kΩ·cm^2^ by the end of the experiment. In contrast, immersion in 40% citric acid resulted in relatively stable |Z|_0_._01_Hz values over 168 h, suggesting persistence of the protective film. Among all tested electrolytes, artificial saliva yielded the highest and most stable |Z|_0.01_Hz values, even during prolonged immersion, indicating superior passive film stability.

The ranking of passive film stability based on impedance measurements decreased in the order: artificial saliva > citric acid > citric–phosphoric acids > H_2_O_2_.

### 3.6. Macro and Micro Structural Aspects

**Figure 7:**
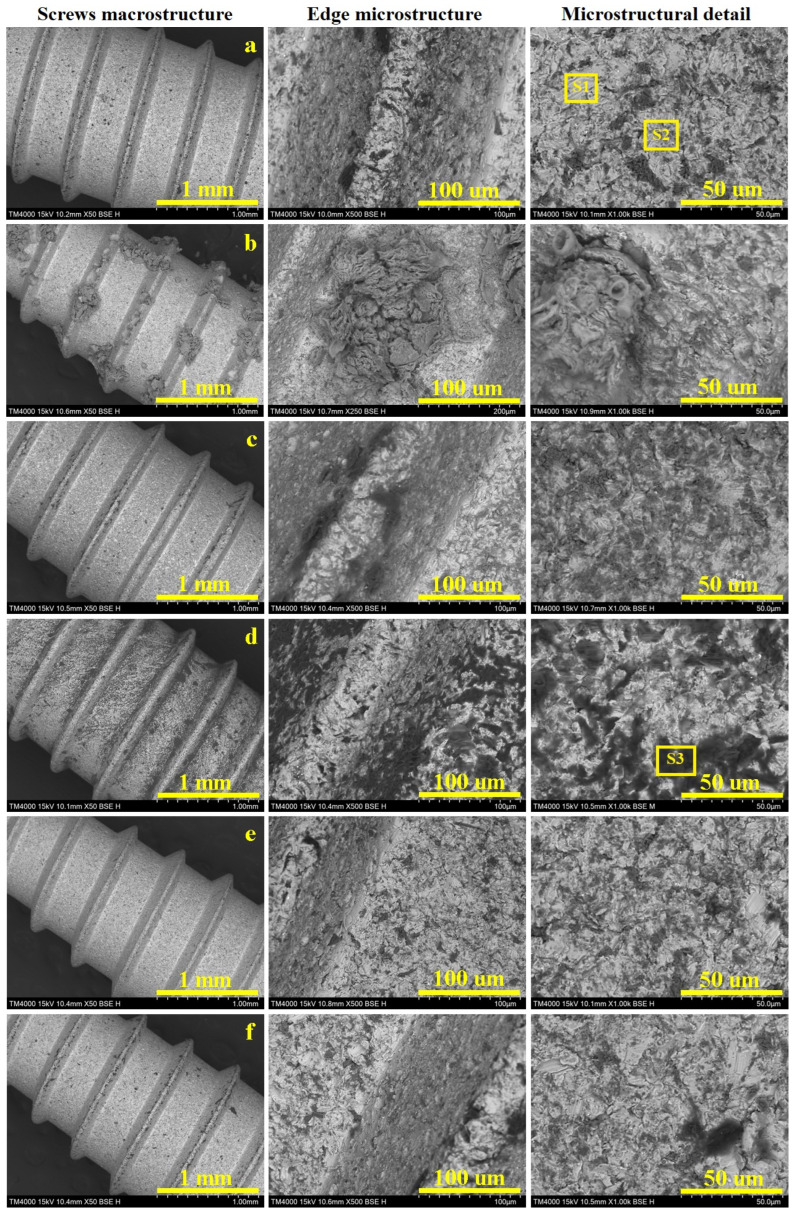
SEM images of the investigated Ti6Al4V screws at macrostructural level with details on the screw edge and grains microstructure: (**a**) control sample, (**b**) Ringer’s solution, (**c**) hydrogen peroxide, (**d**) citric acid, (**e**) artificial saliva, and (**f**) mixture of citric and phosphoric acids.

**Figure 8:**
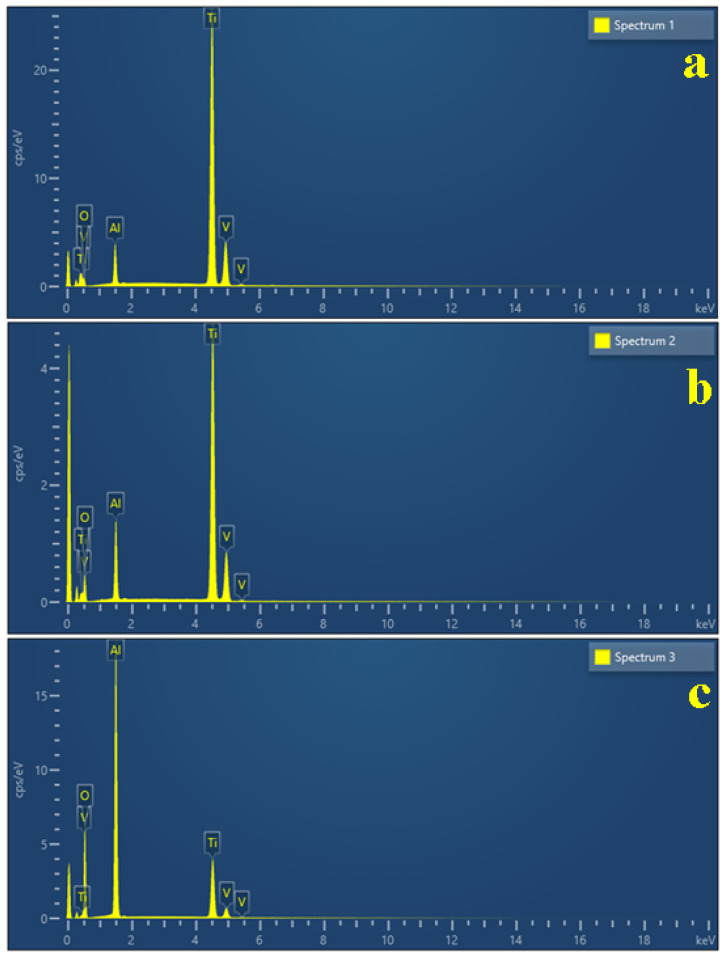
EDS spectra taken for relevant microstructural grains: (**a**) α phase, (**b**) β phase, and (**c**) corroded grain.

Scanning Electron Microscopy (SEM) allows for a proper investigation of both the macrostructural and microstructural aspects which are displayed in Figure 7. The sample’s macrostructure consists in the material organization visible using a 10× magnifying glass and a scale bar of 1–2 mm. Such an observation allows for a proper observation of the screw’s thread consisting in the edges, roots and flanks. Thereafter, the microstructural details were taken at proper magnifications of 500× for the edges and 1000× for the flat surface of the root, allowing for a better observation of the alloy grains. Our samples have polyhedral α grains with a diameter of about 15 µm, tightly embedded with β grains that are about 10 µm in diameter, with internal lamellas with a thickness of about 2 µm (Figure 7a). The grains’ elemental composition was investigated by EDS spectra, taken on their surface spectrum 1 for α grains and spectrum 2 for β grains (Figure 8a,b).

Our measurements reveal that the initial state of the implants have a well-preserved passivated surface, justified by 16.2 wt.% oxygen for α grains, most likely generated by the passivation of Al generating a protective layer of Al_2_O_3_; the lower amount of Al within β grains causes a supplementary oxidation of their surface, justified by the oxygen amount of 31.68 wt.%. Since this is the initial state of the investigated screws, it was mandatory to take an EDS spectrum of the deeply corroded β grain after prolonged exposure to citric acid (Figure 8c), with the oxygen amount increasing up to 52.33 wt.%. Understanding these aspects allows us to better evaluate the morphological changes caused by the prolonged exposure of the screws to the antiseptic solutions (Table 1).

The dental implants control sample has an optimally preserved macrostructure with a smooth edge and root and well-defined flanks of the threaded part (Figure 7a). The flat surface of the edge has a width of about 80 µm, with an optimally preserved microstructure which continues downward on the flanks. The grains aspect is better observed on the thread root, revealing the microstructure previously described as having α grains with a diameter of about 15 µm, tightly embedded with β grains that are about 10 µm in diameter with internal lamellas thickness of about 2 µm. The α grains have nice white aspect, indicating a normal state of the passivation induced by the increased Al content. On the other hand, β grains are slightly oxidized, causing a partial etching of these grains generating a “relative depth contrast”, generating a slightly dark aspect as a consequence of the occurrence of small topographic depressions.

The dental implants immersed in Ringer’s solution (Figure 7b) revealed an optimal preservation of the macrostructure, with precise details of the edge, flanks and root. The optimal activation of the surface has an unexpected effect, consisting of precipitation and subsequently crystallization of the salt minerals, which form macroscopically observable flowers on the thread edge surface. Such a salt crystal flower was observed in the adjacent microstructural detail, revealing a fibrous structure generated by the interlocking of acicular antarctictite (CaCl_2_·6H_2_O), embedded in a compact nest of sylvite (mixed Na and K chloride), crystallized in the cubic system. The detailed morphological observations showed that the salt mineral crystals occur as a superimposed crust that does not penetrate the alloy microstructure, which remains preserved as in the initial state.

Electrochemical measurements revealed that hydrogen peroxide has an intensive corrosive behavior affecting the titanium alloy structure (Figure 7c). The macrostructural aspect looks to be less affected by the prolonged exposure to the hydrogen peroxide, revealing a well-defined edge and root of the thread. Some darker spots appear visible on the flanks. The thread’s edge appear flat and smooth, but the left side reveals deeper dark grains that indicate a highly oxidized state, which is prolonged down on the flanks. The root microstructure reveals that α grains resist well to the corrosive effect, while β grains are prone to be affected by having a darkened aspect. The oxygen excess within this disinfection is effective in killing microorganisms but also promotes the significant oxidation of the implants.

Citric acid proved to be very corrosive, affecting the macrostructure of the investigated screws by generating pitches visible with the naked eye, which become more evident under SEM investigation in Figure 7d. The thread’s edge becomes fuzzy because of the formation of corrosion products which cause it to lose its firmness, while the root looks to be roughened by the corrosion depressions. The microstructural detail indicated that β grains are deeply corroded, generating local depressions with subsequent material loss, which was removed through the corrosive solution. The α grains resist better but they are also affected by darker spots, indicating the passivation layer disruption facilitates the corrosion advance at a longer exposure time.

Artificial saliva is also a mixture of dissolved salts, mimicking the natural human saliva, and therefore, it is a preserving storage medium rather than a disinfection substance. Such an approach makes a difference regarding Ringer’s solution. Thus, the screw’s macrostructure is optimally preserved by prolonged exposure to the artificial saliva (Figure 7e). The microstructural details taken from the thread edge and root reveal that a typical microstructure has a great resemblance to the control sample. It deals with the electrochemical measurements, which indicate a high stability of the passivated layer formed on the screw surface.

The dental implant macrostructure looked well-preserved after prolonged exposure to this acid mixture (Figure 7f). The edge surface is smooth and clear, resembling the control sample. Some corroded β grains occur on the right flanks of the thread and on the root surface. This unexpected observation requires further extensive investigation to reveal the conjugated effect of both acids on the Ti6Al4V alloy. The corrosive effect might be inhibited by the different response of α and β grains to the simultaneous action of both acids hindering each other’s corrosive behavior.

## 4. Discussion

The present study highlights the close interplay between the electrochemical stability of Ti6Al4V surfaces and the corresponding cellular responses following exposure to different chemical treatments that are relevant to dental implant decontamination. The results demonstrate that the dual influences of surface chemistry and topography ultimately determine biocompatibility and long-term implant stability.

The Ti6Al4V is an α + β titanium alloy consisting of two distinct microstructural constituents: α grains with a large polyhedral morphology and compact consistency, and β grains with polyhedral perimeters and lamellar internal structures [12,13]. Elemental analyses consistently show that the α constituent is enriched in Al, while β regions are comparatively enriched in V [14]. This compositional heterogeneity is crucial for corrosion behavior, as a selective attack of β lamellae has been repeatedly associated with electrochemical instability. Interestingly, similar embedding of acicular structures within a compact crust has been described in mineralogical contexts, such as antarctictite crystals in mineral waters [15], suggesting analogous mechanisms of phase-dependent stability.

Electrochemical analyses in the present work revealed electrolyte-specific effects on passivation and corrosion resistance. In 3% H_2_O_2_, the initial ennoblement of the OCP suggested rapid oxide growth, consistent with earlier reports that hydrogen peroxide promotes TiO_2_ film thickening [16,17,18,19,20]. However, the subsequent decline in impedance reflected passive layer destabilization, likely caused by peroxide-induced dissolution of β-phase regions enriched with vanadium, in agreement with previous findings that peroxide radicals compromise Ti–6Al–4V passivity [21,22,23]. This paradox between apparent thermodynamic stabilization (OCP) and kinetic degradation (EIS) confirms that OCP measurements alone may overestimate surface stability, as highlighted in related in vitro corrosion studies [24,25,26].

In citric acid (40%), OCP stabilization and steady impedance values indicated the persistence of a protective oxide film. This aligns with reports that citrate ions stabilize titanium oxide and mitigate dissolution in acidic environments [27,28,29]. By contrast, the citric–phosphoric acid mixture produced progressive impedance reduction, attributable to the aggressive etching action of phosphoric acid [28], which promotes porous film formation and accelerates oxide dissolution [30,31]. Phosphoric acid is a well-known corrosive agent that attacks both carbon steel and aluminum [32,33], and its synergy with citric acid was expected to intensify Ti–6Al–4V corrosion—similar to the way that combined acid exposure accelerates enamel and dentin erosion [34,35]. Artificial saliva yielded the most stable electrochemical performance, consistent with prior studies showing that near-neutral pH and benign salts enhance the hydroxylation and self-healing of passive TiO_2_ films [36,37,38]. Collectively, these results suggest that Ti–6Al–4V implants remain stable in saliva-like environments and citrate-containing solutions but are vulnerable to oxidative or strongly acidic conditions.

The SEM and biological assays corroborated the electrochemical findings by demonstrating how surface alterations translate to cell behavior. The citric–phosphoric acid mixture generated deeply etched and irregular topographies that initially promoted MSC clustering in thread root regions, but simultaneously induced cytotoxicity, reducing viability to below 60%. These effects likely arose from residual corrosion byproducts [39,40] and are consistent with Wennerberg and Albrektsson’s conclusion that contamination may offset the benefits of micro-roughness [39]. Similar cytotoxic responses to residual phosphoric acid have been reported by Xie et al. [41].

In contrast, citric acid alone and Ringer’s solution supported high viability and metabolic activity, aligning with Mombelli et al., who identified Ringer’s solution as a neutral irrigant with low cytotoxicity [42]. Nevertheless, reduced adhesion compared to untreated controls indicated that even mild chemical alterations can disrupt focal adhesion formation, as highlighted by Anil et al. [43]. EDTA-treated surfaces exhibited intermediate cytocompatibility but impaired adhesion, consistent with its known chelation of Ca^2+^/Mg^2+^ ions essential for integrin-mediated attachment, in agreement with Galli et al. [44].

Hydrogen peroxide treatment induced oxidative stress-related damage, with apoptotic features such as membrane disruption and nuclear displacement. These effects corroborate the findings of Liao et al. [45], who reported mitochondrial dysfunction and apoptosis in osteoblasts under H_2_O_2_ exposure. It is also possible that residual hydrogen peroxide remained present in the conditioned medium after the 48 h immersion period, contributing to oxidative stress and the reduced metabolic activity observed in MSCs. Additionally, indirect assays confirmed cytotoxic byproducts in the medium, supporting Ntrouka et al. [46], who cautioned against peroxide-based decontamination due to residual toxicity.

Several limitations should be acknowledged. First, the in vitro environment cannot fully reproduce the complex biological conditions present in vivo, such as continuous saliva flow, dynamic pH fluctuations, mechanical loading, and antibacterial testing, which is highly relevant, given the role of oral bacteria in implant failures. The electrochemical tests were conducted over seven days to assess long-term stability, while cytocompatibility assays were limited to 48 h, due to constraints of in vitro cell viability. This difference should be considered when interpreting results.

Second, only a limited number of chemical treatments and electrolyte compositions were tested; additional physiologically relevant agents, including proteins, enzymes, and inflammatory mediators, may influence corrosion and cytocompatibility. Third, while MSCs were used as a model for initial cellular interactions, longer-term osteogenic differentiation and bone integration processes were not assessed. Another limitation is that only human gingival MSCs were tested. Future studies should also include osteoblasts and oral epithelial cells to better reflect bone and soft tissue responses in the peri-implant environment. Lastly, only Ti–6Al–4V was tested; other titanium alloys or surface-modified implants might exhibit distinct electrochemical and cytological behaviors.

Future studies should aim to bridge the gap between in vitro and in vivo conditions. Long-term dynamic studies that include mechanical loading, fluid flow, and protein/biofilm interactions would provide more clinically relevant insights. Investigating additional alloy compositions, surface coatings, and decontamination protocols could help optimize implant longevity and biocompatibility. For antibacterial evaluation, future studies may employ standardized methods such as those described by Wang and coworkers [47], which could provide reliable protocols for assessing bacterial adhesion and viability. Moreover, the integration of 3D osteogenic cultures or in vivo animal models could clarify how corrosion-induced surface changes influence bone formation, remodeling, and implant integration over time. Finally, advanced analytical techniques could elucidate the chemical composition and nanoscale structural changes in passive films during degradation.

## 5. Conclusions

This comparative study highlights that citric acid and Ringer’s solution uniquely preserve both electrochemical passivity and cytocompatibility, offering new guidance for safer clinical decontamination protocols. Taken together, these findings underscore the balance between surface activation and cytotoxicity. Electrochemical stability strongly influences cellular compatibility: stable passive films in citric acid and saliva promoted favorable biological responses, while destabilizing treatments (H_2_O_2_ and citric–phosphoric mixtures) compromised both passivity and cytocompatibility.

## Figures and Tables

**Table 1:** Elemental composition of the microstructural constituents’ grains.

Microstructural Grain	Spectrum 1	Spectrum 2	Spectrum 3
	α Phase	β Phase	Oxidized Grain
Element	Weight %	Weight %	Weight %
Al	6.88	9.66	32.23
Ti	73.90	55.54	14.72
V	3.10	3.12	0.72
O	16.12	31.68	52.33
Total	100.00	100.00	100.00

## Data Availability

The data presented in this study are available on request from the corresponding authors.
